# Recentering responsible and explainable artificial intelligence research on patients: implications in perinatal psychiatry

**DOI:** 10.3389/fpsyt.2023.1321265

**Published:** 2024-01-18

**Authors:** Meghan Reading Turchioe, Alison Hermann, Natalie C. Benda

**Affiliations:** ^1^School of Nursing, Columbia University School of Nursing, New York, NY, United States; ^2^Department of Psychiatry, Weill Cornell Medicine, New York, NY, United States

**Keywords:** artificial intelligence (AI), explainable AI, bioethics, perinatal psychiatry, patient centered care

## Abstract

In the setting of underdiagnosed and undertreated perinatal depression (PD), Artificial intelligence (AI) solutions are poised to help predict and treat PD. In the near future, perinatal patients may interact with AI during clinical decision-making, in their patient portals, or through AI-powered chatbots delivering psychotherapy. The increase in potential AI applications has led to discussions regarding responsible AI and explainable AI (XAI). Current discussions of RAI, however, are limited in their consideration of the patient as an active participant with AI. Therefore, we propose a patient-centered, rather than a patient-adjacent, approach to RAI and XAI, that identifies autonomy, beneficence, justice, trust, privacy, and transparency as core concepts to uphold for health professionals and patients. We present empirical evidence that these principles are strongly valued by patients. We further suggest possible design solutions that uphold these principles and acknowledge the pressing need for further research about practical applications to uphold these principles.

## 1 Introduction

An estimated one in five women in the United States experience depression between conception and 6 months postpartum (1). For this reason, the United States Preventive Services Task Force recommends routine perinatal depression (PD) screening (2). Other studies have estimated perinatal anxiety may be more prevalent, but there are not yet widely used, perinatal-specific screening tools such as the Edinburgh Postnatal Depression Scale (3). Clinician time constraints, resource availability, validity of screening tools across the perinatal period, and patient willingness to report mental health symptoms present challenges to identification (4, 5). Many also face barriers to treatment including limited access to perinatal psychiatrists and financial constraints (6, 7). As a result, therapeutic interventions are initiated late after symptoms develop (e.g.*, secondary prevention*) (5), and as many as 75% of women who experience symptoms of perinatal mood and anxiety disorders (PMADs) go untreated altogether (8). Left untreated, PMADs can lead to poor maternal and infant outcomes, including pre-eclampsia (9), prematurity (10), low birth weight (11, 12), and behavioral dysregulation (13–15), lower vagal tone, decreased immunity, and adverse neurodevelopmental outcomes in the child (16). Suicide and overdoses are leading causes death in the first year postpartum (17–19).

In the setting of underdiagnosed and undertreated PMADs, novel strategies are needed. Artificial intelligence (AI) is poised to help address these barriers to PMADs diagnosis and treatment. AI involves the development of computer systems that are able to perform tasks that normally require human intelligence such as classifying and predicting disease, recommending treatments, and producing interactive chatbots that leverage large language models to generate humanlike responses (20). For example, AI models can accurately identify women with an elevated risk for PD, enabling primary prevention, before the onset of severe symptoms (4–6). Primary prevention is significantly more effective than secondary prevention for at-risk women (21). Numerous studies have reported on predictive models that can predict PD with high accuracy using a range of datasets (22–24). Specifically, researchers have used electronic health records (EHRs) (25), administrative claims data (26), actively and passively collected data from smartphones (27), social media data (27), and patient-reported outcomes (27, 28), to predict PD, and, in one study, postpartum psychiatric admission. AI-powered chatbots have also become increasingly researched in mental health generally for their potential to deliver psychotherapy (29, 30), and may soon be tailored to the unique needs of perinatal individuals (31, 32).

Artificial intelligence for perinatal psychiatry is now in the early, proof-of-concept stages, but researchers, health systems, and companies are planning for implementation (32). The American College of Obstetrics and Gynecology (ACOG) has also issued guidelines highlighting the potential for AI and other technologies to enhance pregnancy-related care (33). Now is the time to ensure that patient perspectives are understood and integrated into care delivery that uses AI; failure to do so may undermine the very trust in clinicians that is so fundamental in mental healthcare. Research on the bioethical implications of AI in healthcare has raised many important patient safety and quality considerations (34). These conversations have generated popular terms such as *responsible AI (RAI)*, which calls for AI that is ethical, transparent, and accountable while also aligning with stakeholder expectations (35, 36). Similarly, there has been significant interest in *explainable AI (XAI),* which is a set of techniques ensuring that a person can understand how or why an AI system came up with a certain output (37). XAI can be considered a subdomain of RAI because transparency and explainability help identify biases and support autonomous decision-making.

However, RAI and XAI work has primarily assumed that AI was *patient-adjacent:* that patients are recipients of AI-propagated benefits or harms, but that they do not interact directly with it. Rather, we argue that there is a need to equally consider *patient-centered AI*, in which the patient interacts directly with AI during the course of their care. There are many envisioned use cases of patient-centered AI in perinatal psychiatry. Patient engagement and shared decision-making continue to rise in importance in healthcare, making it likely that AI-generated risk predictions will be discussed or presented to patients in clinical care as part of the decision-making process (38). Additionally, new United States policies prohibiting “information blocking” have also led to a deluge of information being returned to patients through patient portals (39), and this information may soon include AI-generated risk predictions that are integrated into EHRs. Finally, AI-powered chatbots delivering psychotherapy represent another example of patients directly interacting with AI (40). Considering patient-centered AI calls for understanding how to responsibly implement AI in a manner that further fosters, rather than undermines, trust. Therefore, in this perspective, we will describe RAI and XAI research, relate it to patient-centered AI, articulate important bioethical concepts in patient-centered RAI and XAI, and describe why these concepts are of particular importance for perinatal psychiatry. We argue that patient-centered AI must consider bioethical constructs while also factoring in important elements of context including type of data being used to develop AI, persons with whom AI or data are shared, what tasks AI supports, and intended time period of use (i.e., preconception, pregnancy, or postpartum).

## 2 The current landscape of RAI and XAI

Many ethical concerns have been raised about the uses of AI in healthcare. From a legal and regulatory standpoint, the European Union (EU) has issued seven key requirements for trustworthy AI, which relate to human agency and oversight, technical safety and rigor, privacy, transparency, fairness, societal and environmental well-being, and algorithm accountability (21). The EU also has strict regulations, the General Data Protection Regulation (GDPR), regarding data sharing and secondary uses of personal data, which relate to AI algorithm development (41). Such guidance and regulations are lacking, however, in the United States and many other nations worldwide, though the White House has proposed the AI Bill of Rights, containing many elements similar to the GDPR (42).

Responsible AI encompasses the conceptual and empirical work investigating responsible uses of AI (36). Four key ethical principles of beneficence, non-maleficence, autonomy, and justice have been adapted to the AI use case, with new acknowledgments of the interplay between these principles and the external agencies, patients, and the complex technical and clinical environments with which AI is used (43). Research has shown that deliberately communicating information about plans to enact these principles when implementing AI in healthcare is associated with favorable attitudes, satisfaction, and high usage intentions among clinicians (36). Some have proposed practical guidelines to operationalize these principles when AI is implemented in clinical practice, including checklists, governance processes, and training for clinicians (44). We have identified that communicating the purpose (why the AI was developed and what it does), process (how it operates), and performance (how well it functions) of an AI system helps users form greater trust (45). In general, however, the majority of RAI work has been highly theoretical, and more practical guidance is needed (46). This research has also primarily considered clinicians to be the end users, while patients are passive participants in the AI–they are considered recipients of the benefits and harms of AI, but not agents interacting directly with it (47–49).

A fifth bioethical principle, explainability, has been proposed as relating to, and upholding, the other four principles previously mentioned (36, 50). Explainability, or XAI, is the idea that humans should have insight into how an AI system works. In the literature, it is an often-used yet poorly defined term. Some have defined it as broadly as technological transparency in general, while others argue it has specific requirements including the ability of a human operator to reconstruct how an AI system uses data, moves through a decision-making process, and arrives at a conclusion (37, 51). XAI has been described as a necessary component of RAI because without explainability, it is difficult for clinicians to exercise their independent clinical judgment (i.e., act autonomously), or for them to ensure that AI is helping, not harming, all of their patients–not just a select few (i.e., upholding beneficence, non-maleficence, and justice) (36). XAI is necessary because of the “black box” problem; for many types of models, particularly those that use deep learning (a type of machine learning), the internal workings of the algorithm are unknown to the developers or end users. Explainability measures attempt to “shine a light” into the black box by using additional algorithms to understand how and why an AI model is producing certain output (52, 53). For example, XAI may tell a clinician which variables included in a model are most contributing to a specific patient’s risk of developing PMADs. To further enhance clinical utility, researchers have also explored visualizations of these explainability measures (54). However, as with RAI research in general, nearly all of the research into XAI techniques considers clinicians to be the primary end users (38). Therefore, the unique information needs and bioethical concerns of patients who may be directly interacting with AI in the course of their care have not been fully considered.

Concepts of RAI and XAI are also complex as perspectives may vary based on context. For example, a recent survey of 610 United States-based adults (sampled to reflect racial, sex, and age demographics of the United States) found that willingness to share health information varied by the type of data (sexual health information, imaging data, genetic data, or mental health information) and persons with whom the information would be shared (e.g., health technology companies, doctors and nurses, and chosen friends and family members). Of note for perinatal psychiatry, participants were least comfortable sharing sexual and mental health information, respectively (55).

## 3 RAI and XAI matter to perinatal psychiatry patients

Trust in AI is one of the biggest challenges in clinical practice and one of the goals of AI implementation has been to foster appropriate trust. Much of the AI implementation research to date has focused on presenting model output and fostering trust in AI among clinicians (45). Maintaining the trust of patients who may be directly interacting with AI is of equal importance, as trust is a fundamental requirement of patient-centered perinatal psychiatry.

Perinatal psychiatry presents a complex bioethical case because of the sensitivity of both pregnancy and mental health data, and the multiple layers of autonomy. In pregnancy, the autonomy, harms, and benefits afforded to the pregnant person, newborn or fetus, and partner must be weighed simultaneously (56, 57). Mental health issues in the perinatal person can negatively impact the newborn or fetus, and partners play an important supportive role in caring for both the perinatal patient experiencing PMADs and the newborn. There is also a layered network of care providers that may at different points in time include, obstetrics, midwifery, doulas, mental health professionals, primary care providers, and pediatricians. Although, the perinatal person may have different levels of comfort disclosing PMADS symptoms to different roles, for example their own obstetrician vs. their child’s pediatrician. Furthermore, perinatal persons may be reticent to accept AI solutions that can predict or treat PMADs. Some concerns relate to the way their personal health data is being used. Numerous research studies have noted that integrating multiple datasets including EHRs, claims data, patient-reported outcomes, and data collected from smartphones and social media improves prediction accuracy (26–28). Perinatal people may be alarmed to discover their EHRs are being repurposed and used for AI-based risk prediction, and may first learn about it when encountering an AI-based risk prediction with their clinicians. Similarly, perinatal persons may have unique concerns about wearable data being hacked or inappropriately shared (e.g., disclosing their location and movements in ways that may be dangerous). For example, many have shed light on the privacy violations and monetization of data collected from period tracking applications (58, 59). These issues are often compounded by the broader concerns about revealing a mental health diagnosis during the perinatal period, such as fear of stigma or loss of custody of one’s baby (22, 60). Moreover, ethical considerations and factors needed to build trust differ by demographic variables, such as race and/or ethnicity, due to well-documented issues of algorithmic bias (61). Therefore, it is imperative that approaches to patient-centered AI for perinatal applications inclusively represent different patient groups, taking special care to ensure minoritized groups may equitably reap benefits and not be disproportionately exposed to harms of AI.

Although in its nascent stages, research on perinatal patients’ attitudes toward AI confirms their strong desire to be involved and informed about how AI is being used in their care. For example, one survey examined the perspectives of 258 English-speaking United States persons registered with an online survey-sampling platform regarding their attitudes toward AI use in mental healthcare. The sample was reflective of the United States population based on race. While the study involved persons with inadequate health literacy (24%), those with less than a Bachelor’s degree (47%), and persons reporting Hispanic/Latino ethnicity (6.5%), these numbers are generally lower than national averages. The authors found most participants reported it was “very important” for them to understand AI output, that participants with a history of pregnancy were significantly more likely to feel this transparency was important, many participants were concerned about medical harm resulting from AI, inappropriate data sharing, and that AI may lead to their mental health provider not knowing them as well (62). Similarly, another study surveyed 150 pregnant persons in Spain from a single hospital. The respondents had somewhat higher rates of tertiary education than the Spanish public (41% in the study vs. 33% nationally). They found that participants strongly endorsed the need for AI to be responsible, trustworthy, useful, and safe, many have privacy concerns, and, importantly, XAI would increase the trust and confidence of participants who were averse to AI being used in their care (63). Importantly, in both studies, participants reported generally high levels of openness to AI being used in one’s healthcare, but all wanted their clinical care team to play a role in ensuring the safe, responsible use of AI (62, 63). These issues should be further evaluated in a sample that is better representative of those that have lower literacy, less education, and a greater proportion of those with Hispanic/Latino ethnicity.

The United States-based study described also, found differences in participants’ comfort with AI based on the task the AI performed. Participants were least comfortable with diagnosis delivery tasks (i.e., AI telling someone they have a mental health condition) and recommending medication. Participants were relatively more comfortable with non-pharmacological treatment recommendations and AI performing mental health assessments (62).

## 4 How can we uphold patient-centered RAI and XAI in perinatal psychiatry?

A practical understanding of patient-centered RAI in perinatal psychiatry is critically needed to support patients in seeking care, receiving care, and maintaining a positive therapeutic alliance with mental health professionals when AI may be involved. As a first step, it is important to identify the bioethical issues of importance to patients that may be operationalized in practice in the future. Previously, our team has synthesized research on RAI generally (64), as well as the ethics of using consumer-generated data (65), AI in psychiatry (20), and maternal health (66, 67) to identify core ethical concepts to be considered in the perinatal psychiatry space.

**Figure 1:**
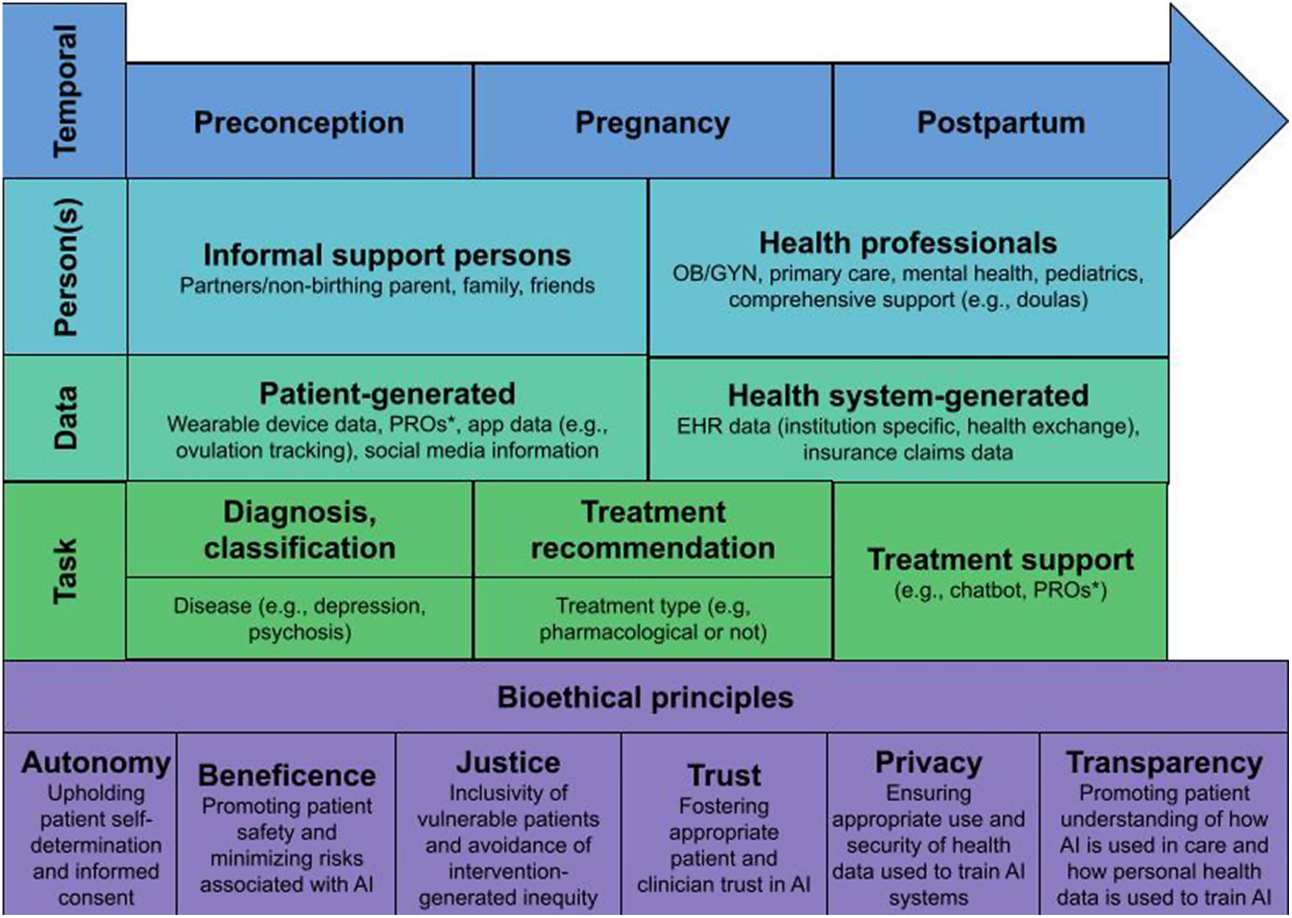
Temporal, person, data, and task-based elements that must be considered in the context of bioethical principles to support RAI and XAI for perinatal psychiatry. * Patient reported outcomes.

We propose six bioethical constructs for upholding patient-centered RAI, including *autonomy, privacy, beneficence, justice, transparency, and trust* (also shown in Figure 1). *Autonomy* calls for patient self-determination regarding how their data are used by those developing AI, and provide informed consent in ways that are specific to the data type, recipient, and use case. We assert that this autonomy lies with the birthing person involving informal support (e.g., partners, family members) as desired, or in rare circumstance where medical proxies may be required. Relatedly, *privacy* calls for the appropriate and confidential uses of personal health data to train AI, and for data use to be deferential to patient wishes as described in a consent process. Communication of privacy-related practices is even more crucial from a reproductive health perspective given the sensitivity of the data that may be involved. When data involve the infant, privacy from the perspective of the non-birthing partner may also become a consideration. *Beneficence* calls for AI to demonstrably improve patient outcomes and, as an implied partner to this, that risks and harms from AI are minimized (i.e., non-maleficence). In perinatal settings, beneficence must not only consider the birthing person but also ensuring potential harms to the child are minimized while prioritizing the autonomy and wellbeing of the birthing person. *Justice* calls for the equal and fair access of the benefits of AI to be evenly distributed among all patients. As part of this, it is important to consider the diversity of patients affected by AI, which includes considering potential sources of algorithmic bias as well as differing information needs and preferences based on different levels of health literacy, numeracy, and experiences of implicit bias in healthcare interactions. Given United States-based disparities in perinatal health outcomes, the disproportionate share of adverse perinatal outcomes occurring in low-and-middle-income countries, and the lack of mental health support for minoritized groups, justice holds particular importance in perinatal psychiatry. *Transparency* calls for XAI that considers the patient as the end user, and ensures that patients can understand how AI is used in their care, how their personal health data was used for that AI, and, crucially, how AI arrived at a specific conclusion relating to their health or healthcare. This principle may be the most different from the general discussions of XAI and RAI as communicating AI output to patients has not previously been explored. Finally, underlying each of these principles is *Trust—*the need to ensure that trust between patients, clinicians, and AI is not undermined. Because AI research both within and beyond perinatal psychiatry has demonstrated that clinicians’ perceptions of AI play a significant role in patients’ attitudes toward it (62, 63, 68, 69), we must consider the interplay of trust between patient, clinician, and AI.

Our empirical research with 258 pregnancy-capable (female sex at birth) United States-based survey respondents (also described above) showed that these concepts resonated with participant groups relevant to perinatal psychiatry. The majority of participants surveyed about the potential use case of AI for mental health endorsed that it was “very important” that they could understand which of their individual risk factors for depression are used by AI (85%), AI will decrease the chance of negative outcomes (77%), they were aware of how their personal data was being used for AI (75%), they were able to make up their own mind about their risk for depression based on AI output (71%), they can understand how likely it is that they develop depression within the next year according to AI (71%), and AI will improve depression and/or depressive symptoms (62%) (62).

The endorsed importance of the six bioethical constructs among survey participants suggests they may serve as a future research agenda for those studying the ethical implications of patients interacting with AI in mental healthcare. However, given the sensitivities and complexities related to perinatal psychiatry, there are also data, person, task, and temporal factors that must be considered. Figure 1 outlines important elements to consider as it pertains to each of these factors in the context of perinatal psychiatry. First, patients may have different needs or perspectives, or there may be different persons to involve based on *temporal* factors, specifically whether they are preconception, currently pregnant, or postpartum. This also considers that patient perspectives and needs are fluid within and outside of these time frames. Second, there are numerous *persons* who may influence data used for AI or may have access to AI-related information, including both informal support persons and health professionals. Which persons are involved and patient perspectives regarding their involvement will likely be individually dependent. Third, AI may be informed using various different kinds of patient and health-system generated *data* elements, some of which have been described. While these elements may not differ much for perinatal persons, the information may have increased sensitivity which should be considered, for example, app-related data that track ovulation or pregnancy, or mental health reported outcomes. Fourth, AI may be used to support diagnosis/classification, treatment recommendation, or treatment support related tasks. Needs and opinions on diagnosis and classification tasks may vary based on perceived severity of disease (e.g., psychosis vs. moderate depression) or other factors. Based on our previous work, patient comfort with treatment recommendation tasks may vary based on whether or not the treatment involves pharmacotherapy, and these perceptions may also differ throughout the preconceptions, pregnancy, and postpartum life-cycle. Fourth, treatment support may come in many different forms with varied levels of autonomy, such as using patient-reported outcomes (PROs) to support health-professional led care to having a nearly-autonomous chatbot leading therapy. Last, these factors must continue to be weighed with the foundation of bioethical principles described above. There are likely more questions than answers related to AI use for perinatal psychiatry in these nascent stages, but we advocate that a research agenda should consider these bioethical constructs and contextual factors in order to ensure AI in perinatal psychiatry may be safe, inclusive, and patient-centered.

It may be possible to design patient education materials, or features in the AI-integrated technology itself, to uphold bioethical principles in practice while still considering important contextual factors. For example, computational interpretability methods that explain important predictors in AI-generated risk prediction [e.g., SHapley Additive exPlanations, or SHAP (70)] may improve patient and clinician trust in the AI and foster its appropriate, safe use. Because visualizations objectively improve comprehension (71, 72), these methods should include visualizations that are comprehended by patients, not just clinicians. Additionally, patient educational materials or “InfoButtons” embedded directly in AI-enhanced technologies can explain an AI’s purpose, its use of personal health data, and its performance, which may support autonomy and transparency. Communication of this information should also follow inclusive design principles to uphold distributive justice (73). Based on described shortcomings in the samples included in previous relevant research (62, 63), it is also important that further empirical studies be conducted with samples that reflect the ethnicity, literacy, and educational backgrounds of diverse perinatal populations. This may be accomplished scientifically by instituting sampling quotas or over-sampling typically under-represented groups. Practically, it will be important to work with community-based organizations to support: recruitment, use of appropriate language, and fostering trust with groups that have a history of facing discrimination in a healthcare setting.

In the near future, perinatal patients may interact with AI during clinical decision-making (38), in their patient portals (39), or through AI-powered chatbots delivering psychotherapy (40). Current discussions of RAI are limited in their consideration of the patient as an active participant with AI. Therefore, we propose a patient-centered, rather than a patient-adjacent, approach to RAI and XAI, that identifies autonomy, beneficence, justice, trust, privacy, and transparency as core concepts to uphold. Although we suggest possible design solutions, research about practical applications to uphold these principles is needed as a next step.

## Data availability statement

The original contributions presented in the study are included in the article/supplementary material, further inquiries can be directed to the corresponding author.

## Author contributions

MT: Conceptualization, Writing – original draft. AH: Writing – review & editing. NB: Conceptualization, Writing – review & editing.
